# Cataract Surgery in One-Eyed Patients: A Cohort Study of 100 Patients

**DOI:** 10.1155/2021/5581512

**Published:** 2021-09-20

**Authors:** Alexis Charles, Pascal Staccini, Arnaud Martel, Stéphanie Baillif

**Affiliations:** ^1^Department of Ophthalmology, Pasteur 2 University Hospital, University of Nice Cote d'Azur, 30 Avenue de la Voie Romaine, 06000 Nice, France; ^2^Department of Statistics, Pasteur 2 University Hospital, University of Nice Cote d'Azur, 30 Avenue de la Voie Romaine, 06000 Nice, France

## Abstract

**Purpose:**

To determine the course and outcomes of cataract surgery in one-eyed patients.

**Methods:**

This retrospective cohort study was conducted at the University Hospital of Nice, France. All one-eyed patients who underwent cataract surgery in their functional eye between January 2014 and December 2018 were included. A one-eyed patient was defined as having a visual acuity (VA) ≤20/200 in the other eye. Data were collected from the medical records and included the sociodemographic factors, the past medical history, data from the preoperative and postoperative clinical examinations, the surgical course, and the visual outcomes.

**Results:**

One hundred one-eyed patients with a mean age of 74.01 years were included (48 men/52 women). The mean preoperative VA was 20/100 (+0.74 logMAR). The VA ranged between 20/200 and 20/40 in 75 (75%) patients, was >20/40 in 8 (8%), and was <20/200 in 17 (17%) patients. Fifty-eight (58%) patients were operated on an outpatient basis. General or locoregional anesthesia was used in 29 (29%) and 9 (9%) patients, respectively. All cataract surgery procedures were performed by phacoemulsification. Five (5%) patients experienced intraoperative complications. Seventy-three (73%) one-eyed patients achieved a final VA ≥20/40. The mean final VA was 20/50 (+0.37 logMAR) (*p* < 0.001).

**Conclusion:**

A low rate of intraoperative complications was observed in one-eyed patients during cataract surgery. In most cases, a good visual recovery was achieved after cataract surgery, even in patients who experienced a surgical complication.

## 1. Introduction

Cataract is one of the leading causes of blindness worldwide [1]. Cataract surgery is one of the most common surgical procedures performed in France with almost 800,000 procedures yearly (according to the PMSI (programme de médicalisation des systèmes d'information) database). It is generally a safe procedure but it can lead to surgical complications, occurring during surgery (intraoperative complications) or in the days or months following surgery (early and late postoperative complications). In addition to the risk of ophthalmic complications, there is also an anesthetic risk when general anesthesia is performed.

A one-eyed patient has only one functional eye, the other being severely visually impaired for various reasons: the ocular loss can be functional and/or anatomical. When one-eyed patients have a cataract in their seeing eye, ophthalmologists face several challenges [2]: would it be better to perform surgery earlier to facilitate the surgical procedure, although there is a risk of operative complications for a slight benefit (benefit-risk ratio)? Or on the contrary, would it be better to wait for a poorer preoperative VA to achieve a greater postoperative benefit, knowing that patients would be exposed to an increased risk of surgical complications? Should we use general anesthesia in these cases to limit the risk of patient movements, or to avoid complications related to retrobulbar/peribulbar anesthesia? What additional precautions should be taken to prevent surgical complications?

This study aimed to determine the course and outcomes of cataract surgery in one-eyed patients, considering the operating conditions, the course of the surgical procedure, the postoperative visual outcomes, and the incidence and evolution of the intra- and postoperative complications.

## 2. Methods

### 2.1. Study Design

A retrospective observational cohort study was conducted in the Ophthalmology Department of Nice Pasteur 2 University Hospital Center between January 2014 and December 2018. This study was conducted in accordance with the principles of the Declaration of Helsinki. The study was approved by the French National Society of Ophthalmology (IRB Number: 00008855).

### 2.2. Eligibility Criteria

All one-eyed patients who underwent cataract surgery in their functional eye between January 2014 and December 2018 were included. Patients were considered “one-eyed” when the Best-Corrected Visual Acuity of the visually impaired eye was ≤20/200 regardless of the etiology (functional or anatomical).

### 2.3. Data Collection

Data were retrospectively collected from the patient charts. Clinical data included the general characteristics of the studied population, the past ophthalmologic and general history, the clinical features of the visually impaired eye, data from the preoperative clinical examination, the surgical procedure, and the postoperative follow-up.

All patients underwent cataract surgery using the ultrasonic phacoemulsification technique (Infinity® phacoemulsifier, Alcon, Texas, United States).

Data from postoperative examinations performed one day (D1), one week (D7), and one month (M1) after surgery were collected. Data of the last ophthalmological examination were also recorded. Early and late postoperative complications were recorded (pseudophakic macular edema, pseudophakic bullous keratopathy, IOL luxation, retinal detachment, and endophthalmitis). The occurrence of posterior capsule opacification (PCO) was also recorded.

### 2.4. Statistical Analysis

Quantitative variables were compared using the nonparametric Kruskal–Wallis test. A value of *p* < 0.05 was considered statistically significant. All VA were converted into a logMAR unit for the statistical analysis. The Kaplan–Meier method was used to assess the time to onset and probability of occurrence of postoperative complications and PCO. Due to the small number of events, no multivariate analysis was performed.

## 3. Results

### 3.1. Demographics of the Population

One hundred one-eyed patients from Nice Pasteur 2 University Hospital were included in the study (100 cataract surgeries). Patient demographics and clinical characteristics are shown in Table 1. The patient's mean age on the day of surgery was 74.01 years (30–93 years). Forty-six (46%) patients were referred to the hospital by a local ophthalmologist for surgery. Most patients lived at home (*n* = 94), only 6 patients lived in a nursing home.

High blood pressure, diabetes, and cognitive disorders were found in 56 (56%), 25 (25%), and 9 (9%) patients, respectively. Thirty-eight out of 99 patients (38.08%) were treated with anticoagulants and/or antiplatelets and 7 out of 48 male patients (14.58%) were treated for prostatic disease.

The medical and surgical ophthalmologic history of the eye planned for surgery is reported in Table 2. Forty-eight (48%) patients had an ophthalmologic history that could impair the final postoperative outcome; 28 (28%) patients had an ophthalmologic history that did impact the final VA.

### 3.2. Clinical Features of the Visually Impaired Eye

Table 3 shows all disorders responsible for the loss of visual acuity of the first eye. The precise etiology of the loss was known for 95 out of the 100 patients (95%): the main disorders were amblyopia (*n* = 24/95, 25.26%), retinal detachment (*n* = 16/95; 16.84%), and age-related macular degeneration (17/95, 17.89%). Seven (7.37%) patients lost their first eye following cataract surgery, with 3 cases of expulsive choroidal hemorrhage, 1 case of pseudophakic retinal detachment, 1 case of acute postoperative endophthalmitis, and 1 case of recalcitrant Irvine Gass syndrome. Nine (9%) eyes had phthisis bulbi, 5 (5%) were eviscerated, and one (1%) was enucleated.

### 3.3. Preoperative Examination

Table 4 summarizes the data from the preoperative examination of the eyes to be operated on.

The mean preoperative VA was 20/100 (+0.74 logMAR). It ranged between 20/200 and 20/40 in 75 (75%) patients, was >20/40 in 8 (8%) patients and <20/200 in 17 (17%) patients. The mean VA of patients living at home was 20/100 (+0.73 logMAR), versus 20/160 (+0.88 logMAR) for patients living in a nursing home, without significant difference (*p*=0.91). The mean preoperative VA of patients with no light perception in the other eye (*n* = 17/100) was 20/160 (+0.93 logMAR), and the VA was about 20/100 (+0.66 logMAR) for the other patients (*p*=0.52).

The type of cataract is detailed in Table 4. The anterior chamber was shallow in 6 (8.57%) patients. The mean axial length of the operated eye was 24.36 mm (range: 19.71–38.16 mm). The quality of preoperative dilation was known for 71 out of the 100 patients. Pupil dilation was good (≥6 mm) in 44/71 (61.97%) eyes. It was insufficient (<6 mm) in 27/71 (38.03%) eyes with 17 (23.94%) eyes with a dilation between 4 and 6 mm, and 10 (14.08%) eyes with a small (<4 mm) pupil size. No patient had zonulolysis or lens subluxation. Four eyes had an opacified cornea that could impede visibility during surgery. Pseudoexfoliation syndrome was found in 8 (10.81%) eyes.

### 3.4. Course of the Surgical Procedure

The surgical data of the operated eye are shown in Table 5. Fifty-eight (58%) and 42 (42%) patients were, respectively, operated on an outpatient and inpatient basis.

General anesthesia was used in 29 (29%) patients and locoregional anesthesia in 9 (9%) patients. The surgeon was a senior physician (board-certified surgeon with more than 5 years of surgical experience after board certification) in 57 (57%) cases and a young physician (board-certified surgeon with less than 5 years of surgical experience after board certification) in 43 (43%) cases.

Capsule staining with trypan blue dye was needed in 16 (16.16%) eyes and iris retractors in 9 (9.09%) patients. No capsular tension ring was used.

All operated one-eyed patients could be implanted at the same time. Implantation was not possible in the capsular bag in 5 (5%) patients: three sulcus implants (one patient with posterior capsular rupture, one patient with dexamethasone implant in the posterior capsule, and one with no identified cause) and 2 iris-claw intraocular lenses (Artisan posterior implant for one patient with capsular rupture and for one patient with zonulolysis).

Five (5/99 : 5.05%) patients experienced intraoperative complications: two had a capsular rupture, one had a capsular lesion without rupture, one had iris laceration, and one had zonulolysis. These 5 patients had a preoperative VA <20/40 and it was <20/200 in 2 of them. Regarding complicated surgeries, the operator was a young physician in 80% of cases. General anesthesia was used in 4 out of the 5 complicated surgeries (80%; *p*=0.024).

Among the 5 patients with intraoperative complications, 2 (40%) had lost their first eye following cataract surgery. Among the 3 patients with intraoperative capsular complication (capsule rupture or tear), none experienced pseudoexfoliation syndrome. No floppy iris was reported in the operative reports of patients treated for prostatic disease (benign prostatic hyperplasia). Complementary anterior vitrectomy was needed in only one (1.01%) patient following posterior capsular rupture. No intraoperative complication required conversion to posterior vitrectomy or subsequent surgery.

### 3.5. Postoperative Outcomes

Ninety-nine (99%, *n* = 99/100) patients attended the D1 follow-up visit. Among the 99 patients, 81 (81.8%) patients reported ocular discomfort after cataract surgery, which was considered as a sharp ocular pain in 3/81 (3.70%) patients. The incision was not sealed (positive Seidel test) in 7.07% (7/99) of cases. All these patients were treated with a bandage contact lens. Anterior chamber inflammation was reported in 78/99 (78.8%) eyes and was graded as severe (aqueous cells at 3+, cyclitic membrane) in 6.41% (5/78 eyes with inflammation). Corneal edema was present in 86/99 (86.9%) eyes and considered as major (3+) in 8.14% (7/86 eyes with corneal edema). The D1 IOP was recorded in 89/99 patients. The mean IOP was 18.69 (range: 7–63) mmHg: 29.21% (26/89 eyes) had an IOP ≥21 mmHg. Among these 26 patients, 16 (61.54%) had preoperative glaucoma.

Ninety-nine percent of patients (*n* = 99) attended the D7 follow-up visit and the mean VA was 20/50 (+0.44 logMAR). Among the eyes with postoperative corneal Seidel on D1, only one eye still had corneal Seidel on D7 (which was sutured on D7). The mean IOP was 14.56 (3–35) mmHg: 8.14% of patients were still hypertonic on D7. The IOP was normalized in 4 (16.67%) patients who had ocular hypertension on D1.

Seventy-nine percent of operated patients (*n* = 79) attended the M1 follow-up visit. The mean VA was 20/40 (+0.31 logMAR). The mean IOP was 13.66 (6–26) mmHg. Five cases (5/79, 6.33%) still had ocular hypertension. Four of these patients (80%) with ocular hypertension at M1 had previously been followed and treated for glaucoma.

One patient experienced postoperative subluxation of sulcus implant, but no secondary surgery was needed. No postoperative acute endophthalmitis was reported.

In our series, 8 (8%) patients experienced late complications with 5 cases of Irvine–Gass syndrome (IGS), one case of rebound inflammation (postoperative treatment was not correctly taken), one case of uncontrolled ocular hypertension, and one case of phthisis bulbi. Phthisis bulbi occurred in a context of recurrent retinal detachment related to familial vitreoretinopathy in the patient who underwent cataract surgery combined with silicone oil removal. Regarding the cases of IGS, the meantime to the clinical diagnosis was 91.4 days (81–114 days, 3 months).

PCO was found in 42 (42%) eyes, with a mean time to onset of 248.6 days (1–1,149 days, 8 months). It occurred in 31.5% (5/16) of cases, after implantation of a hydrophobic IOL, and in 44.05% (37/84) of cases after implantation of a hydrophilic IOL (*p*=0.42).

The mean postoperative follow-up duration, defined as the time between the date of surgery and the date of the last ophthalmological consultation, was 328.1 days (10.77 months).

### 3.6. Final Visual Outcomes

The final VA was 20/50 (+0.37 logMAR). The mean final VA was significantly better than the mean preoperative VA (*p* < 0.001). Seventy-three percent of one-eyed patients had a final VA ≥20/40. Eighty-seven (87%) patients had an improvement in their VA compared to the preoperative examination. For the other 13 patients, 10 (76.92%) had a preoperative ocular history limiting the final visual recovery. The mean final VA was 20/20 (+0.08 logMAR) in patients operated on early (preoperative VA >20/40; *n* = 8). In patients operated on later (preoperative VA <20/200, *n* = 17), the mean final VA was 20/250 (+1.1 logMAR). In patients with no ophthalmologic history (*n* = 24), the mean final VA was 20/25 (+0.13 logMAR), compared to 20/50 (+0.45 logMAR) in patients with a preoperative history (*n* = 76) (*p*=0.014). Patients who experienced an intraoperative complication (*n* = 5) all showed an improvement after surgery, with a postoperative VA greater than the initial VA. Patients with postoperative IGS (*n* = 5) had a mean final VA at 20/25 (+0.14 logMAR) and they all showed an improvement after surgery despite this complication.

## 4. Discussion

Our study described a cohort of 100 one-eyed patients who underwent cataract surgery. To our knowledge, this is the most recent study on this topic since that conducted by Resnikoff et al. in 1988 [1].

The major interest of this work was to provide elements of a response to an anxious clinical situation frequently found in our practice when operating one-eyed patients.

VA loss is currently the main reason to propose cataract surgery [3, 4]. Several teams have investigated the best time to schedule cataract surgery with the best benefit/risk ratio. The arbitrary VA of 20/40 has been commonly accepted as a threshold for the indication of cataract surgery. However, several studies have shown the benefit of earlier surgery (with a preoperative VA >20/40), with good results on the patient's visual quality of life [5, 6]. A literature review by Kessel et al. showed that the preoperative VA is a poor predictor of postoperative visual recovery [7]. Indeed, the final visual outcome after cataract surgery may depend more on the underlying retinal and optic nerve function than on the initial VA. On the other hand, a possible decrease in postoperative visual function may be experienced when surgery is performed late (with a very low preoperative VA) due to more surgical complications [8]. Indeed, a low preoperative VA can be associated with an increased risk of capsular rupture during surgery [9]. In our study, the median and mean preoperative VA were 20/632 (+0.5 logMAR) and 20/100 (+0.74 logMAR), respectively. Few patients were operated on very early (*n* = 8/100) with a preoperative VA >20/40, while most of our patients (*n* = 75/100) were operated on with a preoperative VA ranged between 20/200 and 20/40. Our results were similar to those of a large Swedish national study based on the Swedish National Cataract Register (NCR) including about one million cataract surgeries [10]. This study has shown a mean preoperative VA threshold of 20/200 at the beginning of the study (1992) and of 20/50 at the end of the study (2009), with a decreased number of patients operated with a VA ≤20/200. However, in our study, the patients with total blindness in the worst eye (negative light perception) tended to have a lower preoperative VA than the others (20/160 versus 20/100), but the difference did not reach significance (*p*=0.52). It could be assumed that one-eyed patients might be afraid to undergo cataract surgery in their remaining eye and thus delay surgery as long as they can. Regarding the postoperative visual outcome, a better visual recovery was achieved in patients operated on early with a mean final VA of 20/20 (when the preoperative VA was >20/40) compared to patients operated on late with a mean final VA of 20/250 (when the preoperative VA was <20/200). The intraoperative complication rate was low (5/99, 5.05%), but these 5 patients all had a preoperative VA <20/40. In our study, over a mean follow-up period of 10.77 months, early surgery seemed to be an option to be considered in one-eyed patients as it was associated with better visual recovery and a lower risk of complications.

Regarding the modalities of hospitalization, 42% of patients were hospitalized in a traditional ward. An outpatient setting is often preferred, with good visual outcomes and no significantly higher rates of complications [11, 12]. But, in most of our cases, patients were asked to be hospitalized the night after cataract surgery because they lived far from the hospital (>42 km) or were socially isolated. Since 2018, as the result of health care reforms, overnight stays in our hospital for cataract surgery are more strictly limited, and a much higher number of cataract surgeries are performed in an ambulatory setting. However, the French health care system does not recommend outpatient surgery in case of social isolation or if the patient lives far from the hospital.

General anesthesia and locoregional anesthesia were, respectively, used in 29% and 9% of cases. The use of general anesthesia was decided in patients with cognitive disorders (9 patients), a deforming osteoarticular disease with painful back position (1 patient), nystagmus (3 patients), associated ocular comorbidities (9 patients), and a history of complicated cataract surgery in the other eye (7 patients). Nowadays, local anesthesia is the gold standard for cataract surgery [13] because it is a safe procedure associated with a high level of patient satisfaction. However, general anesthesia may be scheduled for the most difficult cases (patients with cognitive disorders, uncooperative patients, patients with ocular comorbidities). General anesthesia may also be requested by the patients for personal reasons (high level of stress, claustrophobia). However, patients and surgeons should be aware that general anesthesia may be associated with systemic and ocular complications. General anesthesia may cause a loss of Bell's phenomenon (often an involuntary upward movement), which may complicate surgery and lead to intraoperative complications [14]. Another study has shown that general anesthesia could significantly extend the operative time [15].

The intraoperative complication rate found in our study was 5.05% (5/99). Four out of the five complicated surgical procedures were performed by a fully certified but still young physician. It has previously been shown that cataract surgery performed by residents is associated with higher rates of pre-and postoperative complications and of reinterventions [16, 17]. Haripriya et al. published an overall intraoperative complication rate of 0.79% for staff, 1.19% for fellows, 2.06% for residents, and 5% for visiting trainees [17]. Nderitu and Ursell have shown a significantly increased operative time when surgery is performed by a junior surgeon [15]. In the last decade, rates of intraoperative complications after cataract surgery decreased a lot due to improved instrumentation and techniques and training programs [18, 19]. Ravindran et al. found a rate of intraoperative complications for phacoemulsification that fell from 1.3% in 2012 to 0.7% in 2018 [18]. In 2018, Staropoli et al. published a rate of intraoperative complications of 2.4% in the simulator-trained resident group versus 5.1% in the simulator-naïve group [19].

Early postoperative adverse events, such as corneal Seidel and corneal edema, did not affect the final postoperative vision. Corneal Seidel and corneal edema were associated with dense cataract. Late postoperative complications mainly included IGS (5 patients), and despite this, the postoperative VA of all patients was improved. To note, nonsteroidal anti-inflammatory and corticosteroid drops were prescribed to all patients after cataract surgery.

In the literature, a few articles have investigated one-eyed patient condition. To our knowledge, no recent study has been published on cataract surgery in one-eyed patients. The main strengths of our study were the large sample size (*n* = 100), the inclusion of a heterogeneous population representative of the general population. The study's limitations were its monocentric design and the limitation associated with data collection. In our study, the outcomes and complication rates were not compared to those of our 2-eyed patients who had cataract surgery during the same period. This would have provided a better picture of the difficulties encountered in our monocular population.

To conclude, in one-eyed patients, cataract surgery is often required but may be stressful for patients and surgeons. Our results, however, showed a low rate of complications that is comparable to that found in the nonmonophthalmic population. Cataract surgery allowed achieving a significant visual recovery, even when a surgical complication occurred. The absence of visual recovery was mainly related to the preoperative ophthalmologic history and was most often predictable. As the mean final VA was 20/20 (+0.08 logMAR) in patients operated on early (preoperative VA >20/40), and 20/250 (+1.1 logMAR) in patients operated on later (preoperative VA <20/200), cataract surgery in one-eyed patients may not be delayed.

## Figures and Tables

**Table 1:** Demographics of the studied population.

*Age (n* = 100 *patients)*	

Mean; median	74.1; 68.25

*Sex (n* = 100 *patients)*	
Men	48 (48%)
Women	52 (52%)

*Living place* (*n* = 100)	
At home	94 (94%)
In a nursing home	6 (6%)

Patients referred by a local ophthalmologist (*n* = 100)	46 (46%)

*Medical history* (*n* = 100)	
No medical history	28 (28%)
High blood pressure	56 (56%)
Diabetes (type 1 or 2)	25 (25%)
Prostatic disease	7/48 men (14.58%)
Cognitive disorders	9 (9%)
Chronic respiratory disease	13 (13%)
Deforming osteoarticular disease	1 (1%)

*Systemic drugs* (*n* = 99)	
Anticoagulants/antiplatelets	38 (38.38%)
Treatment for benign prostatic hyperplasia	7 (7.07%)

**Table 2:** Ophthalmologic history and ocular treatments of the seeing eye.

Ophthalmologic history and ocular treatments (*n* = 100)	*N* (%)
No past history	24 (24)
Acute anterior uveitis	1 (1)
Corneal disease	
(i) Cornea guttata	1 (1)
(ii) Corneal degeneration, dystrophy	3 (3)
(iii) Pterygium	1 (1)

Ocular hypertension/glaucoma	32 (32)
(i) Open-angle glaucoma	30 (30)
(ii) Closed-angle glaucoma	2 (2)
(iii) Neovascular glaucoma	0 (0)

Age-related macular degeneration (ARMD)	22 (22)

Retinal pathologies (except for ARMD)	
(i) Myopic macular degeneration	13 (13)
(ii) Pseudovitelliform macular dystrophy	3 (3)
(iii) Macular hole/lamellar hole	2 (2)
(iv) Epimacular membrane	7 (7)
(v) Retinal detachment	1 (1)
(vi) Familial retinal dystrophy	2 (2)
(vii) Diabetic retinopathy/diabetic macular edema	7 (7)
(viii) Central retinal vein occlusion	4 (4)
Retrobulbar optic neuritis	1 (1)
Nystagmus	3 (3)

Use of hypotonic drops	32 (32)

Surgical history	7 (7)
(i) Trabeculectomy	3 (3)
(ii) Retinal detachment	1 (1)
(iii) Eyelid surgery	1 (1)
(iv) Refractive surgery	1 (1)

Ophthalmologic history limiting the final visual recovery	48 (48)

**Table 3:** Etiologies responsible for the visual acuity loss of the first eye.

Etiologies responsible for the loss of the first eye (*n* = 95)	*N* (%)
Amblyopia	24 (25.26)
(i) Strabismus	8 (8.42)
(ii) Anisometropia	2 (2.11)
(iii) Etiology not specified	14 (14.73)

Ocular trauma	8 (8.42)
Complicated cataract surgery	7 (7.37)

Corneal degeneration	1 (1.05)

*Glaucoma*	
(i) Closed-angle glaucoma	2 (2.11)
(ii) Open-angle glaucoma	3 (3.16)

Retinal disease	
(i) Myopic macular degeneration	5 (5.26)
(ii) Retinal detachment	16 (16.84)
(iii) Macular hole	1 (1.05)
(iv) Age-related macular degeneration	17 (17.89)
(v) Retinitis pigmentosa	1 (1.05)
(vi) Central retinal artery occlusion	3 (3.16)
(vii) Central retinal vein occlusion	4 (4.21)

Inflammatory optic neuritis	2 (2.11)
Posterior uveitis	1 (1.05)

**Table 4:** Preoperative ophthalmic examination.

Preoperative ophthalmic examination	*N* (%)
*Laterality (n* = 100)	
(i) Left eye	60 (60)
(ii) Right eye	40 (40)

*Preoperative visual acuity (n* = 100)	
(i) <20/200	17 (17)
(ii) Between 20/200 and 20/40	75 (75)
(iii) >20/40	8 (8)

Patients with ocular hypertension (IOP > 21 mmHg) (*n* = 95)	12 (12.63)

*Type of cataract (n* = 100)	
(i) Corticonuclear	63 (63)
(ii) Subcapsular posterior	2 (2)
(iii) Subcapsular posterior and corticonuclear	25 (25)
(iv) Brown cataract	7 (7)
(v) White cataract	3 (3)

Pseudoexfoliation syndrome (*n* = 74)	8 (10.81)

*Pupil dilation (n* = 71)	
(i) Good (>6 mm)	44 (61.97)
(ii) Medium (4–6 mm)	17 (23.94)
(iii) Small (<4 mm)	10 (14.08)

Shallow anterior chamber (<2 mm) (*n* = 70)	6 (8.57)

*Ocular axial length* (mm) *(n* = 100)	
(i) Between 22 and 26 mm	76 (76)
(ii) >26 mm	16 (16)
(iii) <22 mm	8 (8)

IOP: intraocular pressure.

**Table 5:** Course of the surgical procedure.

Course of the surgical procedure	*N* (%)
*Operating conditions*	
Hospitalization (*n* = 100)	
(i) Outpatient	52 (52)
(ii) Inpatient	48 (48)

*Anesthesia (n* = 100)	
(i) Topical	62 (62)
(ii) Locoregional	9 (9)
(iii) General	29 (29)

*Surgeon seniority (n* *=* *100)*	
(i) Senior	57 (57)
(ii) Young	43 (43)

*Surgical material*	
Capsular blue dye (*n* = 99)	16 (16.16)
Iris retractors (*n* = 99)	9 (9.09)

*IOL implantation*	
Location (*n* = 100)	
(i) Capsular bag	95 (95)
(ii) Sulcus	3 (3)
(iii) Iris-claw intraocular lens (posterior artisan)	2 (2)

*Material (n* = 100)	
(i) Hydrophilic acrylic	84 (84)
(ii) Hydrophobic acrylic	16 (16)
Toric IOLs (*n* = 100)	4 (4)

Intraoperative complications (*n* = 99)	5 (5.05)
(i) Capsular rupture	2 (2.02)
(ii) Capsular lesion without rupture	1 (1.01)
(iii) Zonulolysis	1 (1.01)
(iv) Iris laceration	1 (1.01)

Corneal suture at the end of surgery (*n* = 99)	6 (6.06)
Subconjunctival injection of corticosteroids (*n* = 99)	6 (6.06)
Automated anterior vitrectomy (*n* = 99)	1 (1.01)

## Data Availability

The authors indicate that they have full access to all the data in the study and take complete and public responsibility for the integrity of the data and the accuracy of the data analysis.
